# Metabolic Myokines and Adipokines in the Follicular Microenvironment: Implications for Oocyte Competence and IVF Outcomes

**DOI:** 10.3390/ijms27083344

**Published:** 2026-04-08

**Authors:** Charalampos Voros, Fotios Chatzinikolaou, Georgios Papadimas, Ioannis Papapanagiotou, Athanasios Karpouzos, Aristotelis-Marios Koulakmanidis, Diamantis Athanasiou, Kyriakos Bananis, Antonia Athanasiou, Aikaterini Athanasiou, Charalampos Tsimpoukelis, Maria Anastasia Daskalaki, Christina Trakateli, Nana Kojo Koranteng, Nikolaos Thomakos, Panagiotis Antsaklis, Dimitrios Loutradis, Georgios Daskalakis

**Affiliations:** 1 Department of Obstetrics and Gynecology, ‘Alexandra’ General Hospital, National and Kapodistrian University of Athens, 80 Vasilissis Sofias Avenue, 11528 Athens, Greece; 2 Laboratory of Forensic Medicine and Toxicology, School of Medicine, Aristotle University of Thessaloniki, 54124 Athens, Greece; fotischatzin@auth.gr (F.C.); loutradi@otenet.gr (D.L.); 3 Athens Medical School, National and Kapodistrian University of Athens, 15772 Athens, Greece; 4 King’s College Hospitals NHS Foundation Trust, London SE5 9RS, UK; 5 IVF Athens Reproduction Center, 15123 Maroussi, Greece; 6 3rd Department of Internal Medicine, Aristotle University Thessaloniki, 54124 Thessaloniki, Greece; 7 Fertility Institute-Assisted Reproduction Unit, Paster 15, 11528 Athens, Greece

**Keywords:** adipokines, myokines, adiponectin, irisin, asprosin, follicular fluid, granulosa cells, mitochondrial function, oxidative stress, in vitro fertilisation (IVF)

## Abstract

Oocyte competency is a crucial determinant of fertilisation success and the initial development of embryos in assisted reproductive technologies. The metabolic and biochemical environment of the ovarian follicle is crucial for determining oocyte developmental potential, alongside genetic integrity. The follicular microenvironment includes a complex network of signalling chemicals that regulate mitochondrial activity, steroidogenesis, oxidative balance, and cellular energy metabolism. Recently, metabolic hormones originating from adipose tissue and skeletal muscle, namely, adipokines and myokines, have received considerable focus as crucial regulators of ovarian physiology. Adiponectin, irisin, and the recently identified hormone asprosin have emerged as crucial metabolic regulators influencing granulosa cell activity, mitochondrial bioenergetics, insulin signalling pathways, and redox homeostasis inside the follicular niche. Adiponectin mostly provides metabolic protection by activating AMP-activated protein kinase (AMPK) and improving insulin sensitivity, which in turn enhances mitochondrial efficiency and steroidogenic function in granulosa cells. Irisin, derived from the breakdown of fibronectin type III domain-containing protein 5 (FNDC5), aids the developing oocyte by facilitating mitochondrial biogenesis, augmenting oxidative phosphorylation, and altering cellular defence mechanisms against oxidative stress. Conversely, asprosin has been associated with glucogenic signalling, metabolic stress, and probable mitochondrial malfunction, suggesting a possible relationship between systemic metabolic problems and negative reproductive consequences. Clinical and experimental research indicate that the levels of these metabolic regulators in follicular fluid may correlate with ovarian response, oocyte quality, fertilisation rates, and embryo development during in vitro fertilisation cycles. This review consolidates current molecular, cellular, and clinical information, clarifying the pathways by which adipokines and myokines influence follicular metabolism and impact oocyte competency. Understanding the metabolic connections between systemic endocrine signals and the follicular milieu may provide novel indicators for reproductive prognosis and provide new treatment targets to improve assisted reproduction outcomes.

## 1. Introduction

Oocyte competency is a crucial determinant of an individual’s reproductive capability. For fertilisation and embryonic development to occur, meiosis must be executed accurately, and chromosomes must be segregated properly [1]. The development of the cytoplasm is also quite significant. During this phase, organelles are relocated, protein synthesis is regulated, and maternal mRNA is stabilised [2]. Inadequate mitochondrial function may result in fertilisation failure or premature embryonic cessation. Nuclear integrity by itself cannot ensure developmental competence. The biochemical and metabolic conditions inside the follicle have substantial regulatory effects. Ovarian follicular fluid provides the substrates and signalling molecules essential for cellular homeostasis. This environment regulates the equilibrium of redox reactions, energy metabolism, and intracellular signalling [3].

Follicular fluid creates a biochemical environment that circulates around the oocyte and somatic cells. Glucose, fatty acids, amino acids, and growth factors facilitate cellular energy utilisation [4]. Steroid hormones regulate follicular development and intercommunication among endocrine glands. Insulin and insulin-like growth factors influence glucose utilisation and anabolic processes. Alterations in the body’s metabolic status modify the composition of the follicular fluid [5]. Obesity and insulin resistance may disrupt metabolic equilibrium. Such issues may induce increased oxidative stress and diminish mitochondrial efficacy. The development of follicles and steroidogenic function may also be influenced. The capacity for oocyte development depends on the stability of this metabolic environment [6].

Granulosa and cumulus cells continuously sustain the oocyte’s metabolic activity. Connexin proteins form gap junctions that facilitate the transfer of metabolic substrates across cells. Connexin-37 and connexin-43 are crucial for this process to occur. Glucose infiltrates granulosa cells and undergoes catabolism via glycolysis. Subsequently, pyruvate and lactate are transported into the oocyte [7]. Oxidative phosphorylation converts these substrates into ATP. Oocytes possess limited glycolytic capacity and rely on metabolic collaboration with adjacent cells. Granulosa cells also modulate steroidogenesis. Oestradiol and progesterone are synthesised via the conversion of cholesterol into various derivatives. Aromatase is a crucial enzyme that regulates this process. StAR (Steroidogenic Acute Regulatory protein) facilitates the transport of cholesterol into the inner mitochondrial membrane, a crucial stage in steroidogenesis. The successful maturation of oocytes relies on effective contact with somatic cells [8].

The electron transport chain synthesises ATP. Complexes I through IV provide the proton gradient required for ATP synthase to function. Sufficient ATP availability promotes meiotic spindle construction and chromosomal segregation [9]. Mitochondria regulate calcium homeostasis and apoptosis. Under cellular stress, the release of cytochrome c activates pathways reliant on caspases. Nuclear transcription factors are essential for mitochondrial biogenesis. PGC-1α, NRF1, and TFAM regulate mitochondrial replication and functionality. AMP-activated protein kinase monitors intracellular energy levels. Elevated AMP concentrations activate AMPK signalling. The activation of this route facilitates energy restoration and enhances mitochondrial function. Reduced mitochondrial activity adversely affects oocyte quality and embryonic development [10].

Metabolic hormones influence ovarian function by directly interacting with cells. Myokines and adipokines regulate glucose metabolism, mitochondrial function, and steroidogenesis. Adiponectin binds to the AdipoR1 and AdipoR2 receptors. The activation of AMPK and its subsequent signalling pathways augment cellular metabolism. The existence of similar receptors in ovarian tissue signifies direct local actions. Granulosa cells respond to metabolic cues by altering gene expression and enzyme activity. These regulatory mechanisms link systemic metabolic signals to ovarian function [11].

Adiponectin regulates steroidogenesis and cellular sensitivity to insulin. AMP-activated protein kinase monitors intracellular energy levels and is activated under metabolic stress. Upon activation, AMPK promotes catabolic pathways that generate ATP, such as fatty acid oxidation, while inhibiting anabolic activities that consume energy, including lipid and protein synthesis. The downstream consequences differ according to cell type, substrate availability, and the overall metabolic environment. In ovarian cells, AMPK signalling is linked to interactions with other regulatory networks, including AKT and mTOR, consequently influencing glucose metabolism, mitochondrial function, and steroidogenic activity in a context-dependent manner. Enhanced mitochondrial efficiency promotes cellular viability [12,13].

Irisin exerts metabolic effects via modulating pathways that regulate mitochondrial dynamics and cellular energy levels. Irisin is generated through proteolytic cleavage of the transmembrane protein FNDC5. Metabolic stress and PGC-1α activation increase the likelihood of release. Binding to integrin receptors has been proposed to occur in several organs. Following receptor interaction, subsequent signalling cascades are activated [14]. The presence of irisin elevates AMPK phosphorylation. Enhanced AMPK activation promotes the catabolism of fatty acids and the proliferation of mitochondria [15]. Under regulated settings, concentrations of reactive oxygen species decrease. Antioxidant enzymes, such as superoxide dismutase and catalase, may have increased expression. Irisin influences the proliferation and viability of granulosa cells [16]. Variations in irisin concentrations during ovarian stimulation may signify metabolic adaptability. The differences between low and high responders suggest a possible effect on ovarian sensitivity to gonadotropins. The regulation of insulin signalling by Akt phosphorylation may further link irisin to follicular metabolic balance [17].

Asprosin is a glucogenic hormone generated through proteolytic cleavage of fibrillin. It circulates throughout the body and prompts the liver to produce glucose. Cellular interactions with receptors activate cAMP-dependent pathways in metabolic organs. Recent data indicates expression in ovarian cells, including granulosa populations. The activation of protein kinase A and subsequent transcriptional regulators may influence the expression of steroidogenic genes. It has also been proposed that AMPK signals may be altered [18]. Reducing AMPK activity may alter mitochondrial function and lipid catabolism. Increased asprosin levels in metabolic diseases may result in augmented oxidative stress. Insulin signalling pathways may potentially be affected. Alterations in the phosphorylation of insulin receptor substrates and the activation of Akt may influence the metabolic processes of granulosa cells. Alterations in glucose utilisation inside the follicle may influence ATP availability. An energy imbalance may inhibit steroidogenesis and follicular development. The regulation of mitochondrial membrane potential may represent an additional mechanism. Although clinical reproductive data remain limited, the theoretical associations between asprosin, insulin resistance, and ovarian dysfunction are physiologically credible. Further investigation is required to elucidate its function within the follicular milieu and its potential impact on the outcomes of assisted reproduction [19].

Metabolic control is a crucial component of follicular physiology. Cellular metabolism, mitochondrial function, and steroidogenesis remain intricately interconnected. A metabolic imbalance may impede oocyte maturation. Such disruptions may result in reduced fertilisation and compromised embryo development. Assisted reproductive technologies provide a method to examine follicular metabolic control. Identifying metabolic indicators may enhance our ability to forecast IVF outcomes. A more comprehensive knowledge of the signalling mechanisms of adipokines and myokines may facilitate the development of targeted therapies [20].

Available studies have examined the roles of metabolic adipokines and myokines within the follicular milieu. Adiponectin, irisin, and asprosin are the primary subjects of examination. We examine molecular processes and biological pathways. The clinical importance in assisted reproduction is also evaluated. Integrating metabolic and reproductive biology may improve the understanding of oocyte functionality and the efficacy of IVF.

Beyond their individual molecular effects, these signals may contribute to differences in ovarian response and IVF outcomes across metabolic phenotypes. Our review focuses on the underlying pathways and their potential clinical relevance in assisted reproduction.

## 2. Follicular Microenvironment as a Metabolic Regulatory Niche

### 2.1. Cellular Architecture and Metabolic Cooperation Between the Oocyte and Granulosa Cells

The ovarian follicle is a collaborative metabolic organisation. The oocyte remains inside the cumulus cells. The cumulus cells are encircled by mural granulosa cells. Biochemical interactions may occur continuously due to the arrangement of components. Transzonal projections, extensions of cells, traverse the zona pellucida. These projections immediately contact the plasma membrane of the oocyte [21]. At these junctions, connexin proteins link cells. Connexin-37 facilitates communication between oocytes and cumulus cells. Connexin-43 connects granulosa cells inside the follicular compartment. Constructing gap junction channels facilitates the passage of signalling chemicals and metabolites. Transporting pyruvate, amino acids, and nucleotides maintains cellular homeostasis. This network ensures that cyclic AMP levels inside cells remain constant. This signalling continuity regulates the initiation and cessation of meiosis [22].

Functional specialisation is what enables the follicle’s energy metabolism to operate effectively. The oocyte has little glycolytic activity. The expression of phosphofructokinase and lactate dehydrogenase is limited. Granulosa cells mostly use glucose. GLUT1 and GLUT3 transporters facilitate the influx of glucose into granulosa cells [23]. Glycolysis transforms glucose into pyruvate via a sequence of enzyme processes. Key enzymes include phosphofructokinase, pyruvate kinase, and hexokinase. Pyruvate accumulates in granulosa cells prior to translocation into the cytoplasm of the oocyte. Pyruvate traverses connexin channels and monocarboxylate transporters. In the mitochondria, pyruvate dehydrogenase converts pyruvate into acetyl-CoA. Acetyl-CoA is used in the tricarboxylic acid cycle. The electron transport chain functions more efficiently when NADH and FADH_2_ are produced. Oxidative phosphorylation synthesises ATP. Sufficient ATP levels facilitate spindle assembly and chromosomal alignment [24].

Mitochondrial metabolism is a crucial component of this integrated system. Mitochondria use respiratory chain complexes to regulate oxidative phosphorylation. Complex I transfers electrons from NADH to coenzyme Q. Electrons generated from the oxidation of succinate are transferred to Complex II [25]. Electron flow traverses complexes III and IV. Proton gradients exist throughout the inner mitochondrial membrane. This gradient is used by ATP synthase to produce ATP. The potential of the mitochondrial membrane facilitates efficient respiration. ATP generation diminishes when the membrane potential decreases. In the absence of ATP, the formation of meiotic spindles is impeded. In these circumstances, inaccuracies in chromosomal alignment and segregation may arise. The quantity of mitochondrial DNA copies influences metabolic capability. An increase in mitochondrial DNA enhances the efficacy of oxidative metabolism [26].

Amino acid metabolism contributes to the health of oocytes. Granulosa cells use specific transporters to regulate amino acid uptake. LAT1 and ASCT2 are two crucial transporters for neutral amino acids. Amino acids are used in the synthesis of proteins and nucleotides [27]. Glutamine participates in the tricarboxylic acid cycle since it degrades into several metabolites. The synthesis of α-ketoglutarate facilitates mitochondrial oxygen acquisition. Methionine facilitates methylation processes by contributing to the synthesis of S-adenosylmethionine. This pathway is crucial for regulating gene expression via epigenetics. Histone and DNA methylation influence oocyte development [28].

Lipid metabolism is another significant metabolic process. Proteins that transport fatty acids facilitate their entry into granulosa cells. The formation of acyl-CoA activates fatty acids. Carnitine palmitoyltransferase-1 is responsible for the transport of fatty acids into mitochondria. β-oxidation generates acetyl-CoA and reducing equivalents. This compels the electron transport chain to exert more effort. Regulated lipid oxidation is essential for ATP synthesis. Excessive fat accumulation results in lipotoxic intermediates. Ceramide and reactive lipid species may impair mitochondrial activity. Lipotoxic stress may impair the viability and functionality of granulosa cells [29].

Steroid hormones need both mitochondrial and enzymatic functions for their synthesis. The most prolonged phase involves the translocation of cholesterol into mitochondria. The steroidogenic acute regulatory protein facilitates the transport of cholesterol across mitochondrial membranes. CYP11A1 converts cholesterol into pregnenolone [30]. Pregnenolone is the first precursor in the synthesis of progesterone and oestradiol. Aromatase’s CYP19A1 function converts androgens into oestradiol. Oestradiol modulates the proliferation and differentiation of granulosa cells. Oestradiol influences follicular development and oocyte maturation. The expression of steroidogenic enzymes is contingent upon intracellular signalling pathways [31].

Hormonal signalling regulates the energy use of granulosa cells. Follicle-stimulating hormone attaches to its receptor on the membranes of granulosa cells. Activation of adenylate cyclase elevates intracellular cyclic AMP levels. Upon activation of protein kinase A, phosphate groups are appended to transcription factors [32]. This process produces enzymes that enhance steroid efficacy. The activation of insulin receptors also governs the body’s energy usage. Insulin receptor substrate proteins are activated upon insulin binding. The phosphoinositide-3 kinase and Akt signalling pathways are activated subsequent to receptor stimulation. Relocating glucose transporters facilitates cellular glucose uptake. Cells may enhance energy production when glucose metabolism is optimised [33].

Cells need a proper oxidative equilibrium to function well. Reactive oxygen species are generated during oxidative phosphorylation. Cells may communicate more effectively when they produce substances in a regulated manner. Excessive production is detrimental to DNA and proteins. Antioxidant systems maintain redox equilibrium. Superoxide dismutase converts superoxide into hydrogen peroxide. Glutathione peroxidase and catalase are enzymes that decompose hydrogen peroxide. Glutathione assists cells in maintaining their redox equilibrium. For mitochondrial health, antioxidants must function effectively. Disruption of redox equilibrium hinders granulosa cell functionality and oocyte survival [34].

The follicle remains stable due to the collaboration between granulosa cells and the oocyte. Substrate transfer facilitates mitochondrial respiration. The development of follicles is regulated by the synthesis of steroid hormones. Intracellular signalling channels govern metabolic and endocrine responses. The integrity of mitochondria facilitates energy generation and advances meiotic development. The disruption of this metabolic network diminishes the developmental capacity of oocytes. A deeper understanding of these molecular pathways may improve results in assisted reproduction. While these pathways are comprehensively known at the molecular level, most information is obtained from experiments or research performed on animals. Direct validation in human IVF scenarios is limited, and the degree to which these pathways correspond with clinically relevant outcomes is not well delineated.

### 2.2. Mitochondrial Function and Bioenergetic Regulation in the Follicular Microenvironment

Mitochondria serve as the primary source of ATP for both the oocyte and the surrounding granulosa cells. Oxidative phosphorylation is the process by which energy is generated. This occurs along the inner mitochondrial membrane. This mechanism is regulated by the complexes of the electron transport chain [35]. Complex I receives electrons from NADH produced during the tricarboxylic acid cycle. Complex II donates electrons derived from the oxidation of succinate. Electron transport persists via coenzyme Q and complex III. Cytochrome c donates electrons to complex IV. Oxygen is the last electron acceptor. The movement of protons across the inner mitochondrial membrane generates an electrochemical gradient. This gradient is used by ATP synthase to synthesise ATP from ADP and inorganic phosphate. Ample ATP availability promotes spindle formation and chromosomal integrity during meiosis.

Mitochondrial biogenesis regulates the quantity and functionality of mitochondria. This mechanism is regulated by nuclear transcription factors. Peroxisome proliferator-activated receptor gamma coactivator 1 alpha serves as a crucial regulator. PGC-1α activates nuclear respiratory factor 1 and nuclear respiratory factor 2 [36]. These transcription factors regulate the synthesis of mitochondrial proteins from nuclear DNA. The transcription factor for mitochondria A facilitates the replication of mitochondrial DNA. An increased quantity of mitochondrial DNA enhances respiratory capacity. Enhanced electron transport efficiency results in increased ATP production. Reduced mitochondrial biogenesis results in diminished energy availability for cells. Insufficient energy disrupts meiotic development and cytoskeletal structure [37].

AMP-activated protein kinase functions as a principal energy sensor. During energy stress, elevated AMP levels activate AMPK. Activation occurs due to phosphorylation at the threonine-172 residue. Liver kinase B1 and calcium/calmodulin-dependent protein kinase kinase mediate this phosphorylation. Activation of AMPK accelerates catabolic processes that generate ATP [38]. Upon activation of AMPK, fatty acid oxidation increases. The translocation of glucose transporters facilitates cellular glucose uptake. Inhibiting anabolic processes conserves energy inside cells. Activation of AMPK results in a reduction in mTOR signalling. Reduced mTOR activity diminishes protein synthesis and cellular development. This reaction restores energy equilibrium when the body experiences metabolic stress [39].

Mitochondrial dynamics govern the morphology and functionality of mitochondria. Mitochondrial health is maintained by fusion and fission mechanisms. Mitofusin 1 and mitofusin 2 regulate the fusing of the outer mitochondrial membrane. Optic atrophy protein 1 regulates the fusing of the inner membrane. Fusion facilitates the flow of mitochondrial material and maintains stability. Fission facilitates the removal of damaged mitochondrial components. Dynamin-related protein 1 facilitates mitochondrial fission. Achieving the optimal equilibrium between fusion and fission sustains mitochondrial function. Excessive fission leads to the fragmentation of mitochondria. Fragmentation diminishes ATP synthesis and elevates oxidative stress [40].

The mitochondrial membrane potential indicates the functional efficacy of the mitochondria. The disparity in proton concentration across the inner membrane generates membrane potential. A decrease in membrane potential indicates malfunctioning mitochondria. A diminished membrane potential impedes ATP synthesis. Cytochrome c release may occur when mitochondria are severely compromised. Caspase activation occurs subsequent to the release of cytochrome c. Consequently, apoptotic pathways are activated. The well-being of mitochondria is crucial for the viability of granulosa cells. Impaired mitochondrial function diminishes steroidogenesis and metabolic activity [40].

Oxidative phosphorylation leads to the formation of reactive oxygen species. Superoxide radicals are generated when electrons escape from complexes I and III. Superoxide dismutase converts superoxide into hydrogen peroxide. The enzymes catalase and glutathione peroxidase decompose hydrogen peroxide [41]. Regulated reactive oxygen species generation is crucial to intercellular signalling. Excessive ROS damage mitochondrial DNA, proteins, and lipids. Oxidative damage impairs the efficiency of the electron transport chain. Following oxidative damage, ATP synthesis diminishes. The competency of oocytes depends on the maintenance of mitochondrial integrity [42].

The role of mitochondria influences the synthesis of steroid hormones. The preliminary stage of steroidogenesis involves the translocation of cholesterol into mitochondria. This transport is regulated by steroidogenic acute regulatory protein. CYP11A1 converts cholesterol into pregnenolone inside the mitochondria. Pregnenolone serves as the precursor to subsequent steroid hormones. Mitochondrial malfunction impedes this enzymatic process. Reduced synthesis of steroid hormones impairs follicular development and inter-glandular communication within the endocrine system [43].

Metabolic regulators influence mitochondrial function inside the follicle. Adiponectin activates AMPK signalling and enhances mitochondrial function. Irisin enhances the expression of PGC-1α and promotes mitochondrial proliferation. Asprosin may alter mitochondrial function by influencing insulin signalling pathways. Variations in metabolic hormone levels influence mitochondrial functionality [44]. Alterations in mitochondrial function influence oocyte performance and the outcomes of in vitro fertilisation. The integrity of mitochondria is a vital determinant of reproductive success. Adequate energy generation promotes meiotic advancement and fertilisation. Proper mitochondrial control sustains steroidogenic activity and cellular viability. Disruption of mitochondrial metabolism impedes follicular growth. Comprehending mitochondrial function may elucidate oocyte competence and the results of assisted reproduction [45].

### 2.3. Oxidative Stress, Redox Regulation, and Metabolic Stability in the Follicular Environment

Reactive oxygen species are continuously generated inside the ovarian follicle. The primary source is mitochondrial oxidative phosphorylation. Electrons mostly escape from complexes I and III of the electron transport chain. Insufficient electron transport results in the formation of superoxide anions [46]. Superoxide dismutase converts superoxide into hydrogen peroxide. Hydrogen peroxide traverses many cellular compartments. Catalase and glutathione peroxidase convert hydrogen peroxide into water. This enzymatic activity prevents the accumulation of harmful reactive species. Regulated reactive oxygen species generation is implicated in intracellular signalling. Excessive buildup disrupts cellular equilibrium [47].

Granulosa cells possess robust mechanisms that safeguard them from free radicals. Glutathione is a crucial intracellular antioxidant. Reduced glutathione neutralises reactive oxygen species via redox processes. The process is accelerated by glutathione peroxidase. Following detoxification, oxidised glutathione is produced [48]. Glutathione reductase utilises NADPH to regenerate reduced glutathione to its native state. This cycle maintains the redox equilibrium inside cells. Optimal glutathione levels facilitate normal mitochondrial function. Reduced glutathione depletion increases oxidative stress. Oxidative stress diminishes mitochondrial respiratory efficiency [49].

The NRF2 signalling system regulates the production of genes that safeguard cells against oxidative damage. NRF2 remains inactive when not used due to its binding with KEAP1. KEAP1 facilitates the proteasomal degradation of NRF2. Oxidative stress modifies the structure of KEAP1 by cysteine oxidation. Subsequent to this alteration, NRF2 dissociates from KEAP1. Unbound NRF2 translocates to the nucleus. NRF2 binds to antioxidant response sites inside the promoters of target genes [50]. Consequently, the expression of antioxidant enzymes increases. The concentrations of superoxide dismutase, glutathione peroxidase, and catalase increase. Increased activity of antioxidant enzymes protects cells from oxidative damage. Oxidative stress directly affects the stability of mitochondrial DNA. Histone proteins that safeguard DNA are absent in mitochondria. This increases susceptibility to oxidative damage. Damage to mitochondrial DNA impairs the synthesis of proteins within the respiratory chain. Following mitochondrial DNA damage, the efficacy of electron transport diminishes. Under these circumstances, ATP synthesis diminishes. Energy shortage affects meiotic development and spindle assembly. Consequently, chromosomal instability may occur [51].

A regulated redox equilibrium is essential for granulosa cells to synthesise steroids. Reactive oxygen species influence the enzymatic activity involved in steroidogenesis. Excessive ROS proteins diminish the amounts of aromatase and StAR. The transfer of cholesterol into mitochondria is impeded. During oxidative stress, the body synthesises reduced levels of pregnenolone [52]. Consequently, the body produces diminished levels of oestradiol. Reduced oestradiol levels impede follicular development. An imbalance in hormones may influence the maturation of oocytes. Insulin signalling also influences oxidative stress pathways. Hyperinsulinemia increases the influx of mitochondrial substrates. Increased electron transport activity results in heightened ROS generation. Oxidative stress impairs insulin receptor signalling. The phosphorylation of proteins that serve as substrates for insulin receptors is impaired. Consequently, Akt signalling activity diminishes. Reduced glucose absorption results in diminished energy availability for cells. Granulosa cells exhibit dysfunction in conditions of metabolic instability [53].

Hormones that govern metabolism influence cellular redox regulation. Adiponectin activates AMPK and enhances the expression of antioxidant enzymes. Increased AMPK activity enhances mitochondrial function. Irisin enhances mitochondrial proliferation and reduces oxidative stress. Following irisin signalling, the concentrations of antioxidant enzymes increase. Asprosin may influence oxidative equilibrium via insulin signalling pathways. Elevated asprosin levels have been associated with situations that induce metabolic stress. Alterations in glucose utilisation by the organism may result in increased production of reactive oxygen species by mitochondria.

The redox balance is crucial for oocyte health. Regulated ROS generation enhances intracellular signalling and meiotic control. Excessive oxidative stress damages the integrity of mitochondria and DNA. Oxidative circumstances impede cellular energy production. Reduced ATP availability impedes meiosis. Fertilisation and embryonic development may be affected [54]. Maintaining the functionality of antioxidant defence mechanisms is crucial for the health of infants. Metabolic diseases are associated with issues in redox regulation. Obesity and insulin resistance increase oxidative stress levels. The altered composition of follicular fluid indicates this imbalance. Increased oxidative stress has been associated with decreased success rates in IVF. Compromised mitochondrial function results in reduced oocyte quality. Understanding the control of oxidative stress may improve metabolic assessment in assisted reproduction [55]. Multiple signalling pathways involved are common across numerous metabolic regulators, thereby limiting the ability to attribute a direct causal effect of a specific adipokine or myokine on oocyte competency based on current findings. Table 1 summarises the main clinical studies, including study design, sample characteristics, and primary outcomes, in order to facilitate interpretation of their clinical relevance.

## 3. Adipokines in Follicular Physiology and Metabolic Regulation

### 3.1. Adiponectin Signalling and Molecular Regulation in Granulosa Cells

Adiponectin is a peptide hormone mostly synthesised by adipocytes. It traverses the body in several molecular forms, including low-molecular-weight, medium-molecular-weight, and high-molecular-weight multimers. The high-molecular-weight variety has the greatest biological activity in metabolic organs [61]. Adiponectin infiltrates the ovarian follicle via systemic circulation. Quantifiable quantities have been identified in follicular fluid during assisted reproductive cycles. Adiponectin receptors have been demonstrated to exist in granulosa and cumulus cells in the region. The expression of this receptor promotes direct cellular interactions within the follicular environment [62].

Adiponectin exerts biological effects via binding to the AdipoR1 and AdipoR2 receptors. AdipoR1 is prominently expressed in metabolically active tissues. AdipoR2 is associated with lipid metabolism and mitochondrial regulation. Both receptors include inherent ceramidase activity. The breakdown of ceramide produces sphingosine and sphingosine-1-phosphate. Reducing ceramide levels inside cells enhances insulin efficacy and improves mitochondrial function. The accumulation of ceramide is associated with mitochondrial dysfunction and cellular apoptosis. Activating the adiponectin receptor safeguards the stability of cellular metabolism [63].

AMP-activated protein kinase is a major target of adiponectin signalling. Activation of AdipoR1 induces the phosphorylation of AMPK at threonine-172. When AMPK is activated, it promotes catabolic metabolic pathways that generate ATP. The phosphorylation of acetyl-CoA carboxylase enhances fatty acid oxidation. Inhibiting acetyl-CoA carboxylase reduces the levels of malonyl-CoA [64]. Reduced concentrations of malonyl-CoA inhibit the activity of carnitine palmitoyltransferase-1. Increased CPT1 activity facilitates the entry of fatty acids into mitochondria. β-oxidation enhances the synthesis of acetyl-CoA inside mitochondria. An increased concentration of acetyl-CoA enhances the efficiency of the tricarboxylic acid cycle and facilitates ATP production [29]. AMPK has several isoforms, mostly consisting of the catalytic α-subunit AMPKα1 and AMPKα2. These isoforms exhibit distinct involvement across many tissues and activities. AMPKα1 is mostly present in proliferative and somatic cells, such as granulosa cells. AMPKα2, however, is more intimately associated with organs exhibiting elevated oxidative metabolism. This isoform-specific distribution may result in distinct control of metabolic and mitochondrial activities within the follicular milieu.

Adiponectin signalling also influences mitochondrial biogenesis. Activation of AMPK increases the production of PGC-1α, which functions as a transcriptional coactivator for mitochondrial genes. This route activates nuclear respiratory factors NRF1 and NRF2. Mitochondrial transcription factor A regulates the replication of mitochondrial DNA. An increased quantity of mitochondrial DNA facilitates respiration. Increased activity of electron transport chains results in enhanced ATP production. Enhanced mitochondrial efficiency aids granulosa cells in metabolising nutrients [65].

Adiponectin also alters the functionality of insulin signalling pathways. Adiponectin promotes the phosphorylation of insulin receptor substrate proteins. Phosphoinositide-3 kinase is activated subsequent to receptor signalling. Akt phosphorylation facilitates the translocation of glucose transporters to the cell membrane. Increased glucose absorption enhances energy availability to cells [66]. Enhanced glucose metabolism supports the viability and functionality of granulosa cells. Improved insulin sensitivity reduces metabolic stress in the follicular milieu.

Adiponectin signalling significantly influences steroidogenesis. The transport of cholesterol into mitochondria depends on the expression of steroidogenic acute regulatory protein [23]. Adiponectin regulates the production of the StAR protein via the activation of AMPK. Increased StAR expression enhances the availability of cholesterol for steroidogenesis. CYP11A1 converts cholesterol into pregnenolone inside the mitochondria. Pregnenolone is the precursor of progesterone and oestradiol. Aromatase activity regulates the conversion of androgens into oestradiol. Hormonal and metabolic signalling influence the production of CYP19A1. Adiponectin modulates aromatase expression by intercellular signalling [67].

Adiponectin regulates pathways that promote cellular survival. Activation of AMPK inhibits pro-apoptotic signalling. Reducing caspase activation aids in the survival of granulosa cells. Adiponectin signalling preserves the integrity of the mitochondrial membrane. The release of cytochrome c is constrained under these circumstances. Enhanced mitochondrial stability facilitates ATP production [68]. A sufficient energy source supports steroidogenic activity. Adiponectin also contributes to the regulation of oxidative stress. Activation of AMPK magnifies the activity of antioxidant enzymes. Following adiponectin signalling, the activity of superoxide dismutase and catalase increases. Reducing reactive oxygen species safeguards mitochondrial components. Preserving the integrity of mitochondrial DNA sustains respiratory activity. An improved redox equilibrium contributes to the stability of follicles [44].

The concentration of adiponectin in follicular fluid indicates the efficacy of bodily functions and hormonal activity. Reduced adiponectin levels have been seen in metabolic diseases such as obesity and insulin resistance. Alterations in adiponectin signalling may impair the metabolism of granulosa cells. Reduced adiponectin availability may result in decreased mitochondrial function. The steroidogenic function may be impaired. These modifications may affect oocyte developmental competency. Clinical investigations have shown associations between adiponectin levels in follicular fluid and results of IVF. Increased adiponectin levels are correlated with improved fertilisation rates. Improved metabolic conditions inside the follicle may clarify this link. Enhanced mitochondrial activity facilitates oocyte maturation. Enhanced steroidogenesis facilitates follicular growth. Adiponectin may serve as a metabolic regulator inside the follicular microenvironment [69].

Adiponectin signalling integrates metabolic and reproductive processes. Mitochondrial activity, insulin signalling, and steroidogenesis are regulated by many mechanisms. Preserving metabolic stability facilitates the functionality of granulosa cells. Maintaining mitochondrial health enhances oocyte functionality. Understanding adiponectin signalling pathways may improve metabolic assessment in assisted reproduction.

### 3.2. Adiponectin in Follicular Fluid and Its Association with IVF Outcomes

Adiponectin is consistently present in follicular fluid throughout controlled ovarian stimulation cycles. The concentrations vary across follicles from the same subject. Variations may result from discrepancies in metabolic activity and granulosa cell functionality in the region. Follicular adiponectin concentrations only partly correlate with serum levels in circulation. Discussions have emerged on local production inside granulosa cells. Receptor expression in the follicular compartment enables autocrine and paracrine signalling [70].

Numerous clinical studies have evaluated the relationship between follicular fluid adiponectin and oocyte quality. Elevated adiponectin levels have been associated with increased rates of normal fertilisation. Mature oocytes derived from follicles with increased adiponectin levels often demonstrate improved developmental advancement. For fertilisation to occur, the cytoplasm must undergo appropriate maturation [13]. Cytoplasmic maturation depends on ATP availability and the integrity of mitochondria. Adiponectin-induced AMPK activation may improve mitochondrial activity in cumulus cells. Increased mitochondrial activity enhances pyruvate acquisition by the oocyte. Increased ATP synthesis facilitates the completion of meiosis and the formation of pronuclei [71].

The metabolic composition of follicular fluid seems to influence embryo development. The first cleavage phases need sustained mitochondrial activity derived from the oocyte. Reduced mitochondrial function may impede blastocyst formation. In some cohorts, elevated adiponectin levels in follicular fluid have been associated with improved embryo quality ratings. An improved redox balance in the follicle may prevent oxidative damage to mitochondrial DNA. Reduced oxidative stress contributes to the stability of the cytoskeleton and spindle apparatus [13].

Adiponectin signalling may influence the ovarian response to stimulation. Women with metabolic disorders may have varying amounts of adiponectin. Insulin resistance and obesity are associated with decreased concentrations of adiponectin in the bloodstream. Reduced levels of follicular adiponectin may indicate dysfunction in the ovary’s metabolic signalling. Insulin resistance alters the signalling of PI3K/Akt in granulosa cells [72]. Reduced insulin sensitivity may impede glucose entry into cells. Insufficient glucose impairs granulosa cells’ glycolytic production. A reduced influx of pyruvate to the oocyte may diminish the efficacy of mitochondrial respiration. Deficiencies in mitochondrial respiration impair ATP-dependent mechanisms essential for oocyte maturation.

Adiponectin also influences the signalling of gonadotropins. Follicle-stimulating hormone promotes steroidogenesis via cAMP-dependent mechanisms. Adiponectin may modulate this response by activating AMPK. The activation of AMPK influences transcription factors that regulate the synthesis of CYP19A1 [69]. Altered aromatase activity influences the production of oestradiol. The concentration of oestradiol in the follicular milieu influences the rate of granulosa cell proliferation. Hormonal imbalance may influence the kinetics of follicular development.

Researchers have examined the concentrations of adiponectin in the follicular fluid of women diagnosed with polycystic ovarian syndrome. PCOS is characterised by insulin resistance and alterations in the body’s metabolic signalling mechanisms. Under this scenario, adiponectin levels are often reduced compared to the usual. A deficiency of adiponectin may impede the metabolic regulation of granulosa cells. Reduced activation of AMPK may impede the catabolism of fatty acids. The accumulation of lipid intermediates may occur. Lipotoxic stress may diminish mitochondrial efficiency. It is also conceivable that steroidogenesis may alter. These processes may explain the reduced oocyte quality in metabolically compromised individuals [73].

Researchers have investigated the correlation between adiponectin levels and implantation rates in smaller cohorts. For optimal embryonic development, the oocyte’s cytoplasm must undergo normal maturation. Enhanced metabolic conditions inside the follicle may indirectly influence the embryo’s implantation capability. Consistent mitochondrial inheritance and less oxidative damage facilitate healthy embryonic development. Certain research indicate that elevated adiponectin levels correlate with increased clinical pregnancy rates. However, the outcomes remain disparate across various demographic groupings [74].

When analysing clinical data, it is important to exercise caution about confounding factors. The metabolic composition of follicles is influenced by body mass index, insulin resistance, stimulation regimen, and ovarian reserve. Various methods of quantifying adiponectin may alter the reported associations. Enzyme-linked immunosorbent tests differ in sensitivity and specificity. Various molecular forms of adiponectin may have distinct effects on organisms. High-molecular-weight adiponectin seems to have a more pronounced influence on metabolism. Few studies have examined the impact of various isoforms in follicular fluid. The available information indicates that adiponectin maintains metabolic equilibrium in the follicular milieu. Activation of AMPK signalling enhances mitochondrial function and accelerates lipid metabolism. Enhanced mitochondrial performance facilitates ATP production and maintains redox equilibrium. Regulating steroidogenesis ensures that the endocrine system adequately supports follicular development. Clinical correlations between fertilisation and embryonic development support the notion that it may serve as a biomarker.

However, clinical findings remain inconsistent. Several studies have reported associations between higher adiponectin levels and improved fertilisation rates or embryo quality [13], whereas others have not demonstrated a clear relationship with pregnancy outcomes [56].

### 3.3. Molecular Interaction Between Adiponectin, Insulin Signalling, and Steroidogenesis

Granulosa cells simultaneously respond to insulin and gonadotropins. Insulin regulates glucose uptake and anabolic metabolism. Follicle-stimulating hormone regulates the synthesis of steroid hormones. Both signalling mechanisms converge on the transcriptional control of steroidogenic enzymes. The magnitude and orientation of this convergence are influenced by metabolic condition. In this context, adiponectin functions as a metabolic “buffer”. Adiponectin signalling enhances insulin sensitivity and reduces metabolic stress [69].

The insulin receptor is the initiation point for insulin signalling. Subsequent to ligand binding, the receptor undergoes autophosphorylation. Subsequently, the proteins constituting the insulin receptor substrate undergo phosphorylation. In granulosa cells, IRS-1 and IRS-2 serve as crucial adaptor molecules [75]. Phosphoinositide-3 kinase is recruited by the tyrosine phosphorylation of IRS proteins. At the plasma membrane, PI3K converts PIP2 into PIP3. PIP3 recruits PDK1 and Akt. Subsequently, Akt undergoes phosphorylation and is activated. When Akt is activated, it facilitates glucose uptake by regulating the mobility of glucose transporters. Akt also promotes cell survival by increasing glycolytic flux [76].

Insulin signalling interacts with transcriptional regulators that modify the function of granulosa cells. FOXO1 is a crucial target of Akt. Akt phosphorylates FOXO1, hence preventing its translocation to the nucleus. Nuclear FOXO1 facilitates a transcriptional pathway that adjusts to stress [77]. Prolonged FOXO1 activation diminishes proliferative signalling and alters steroidogenic characteristics. Akt-mediated suppression of FOXO1 enhances the proliferation and functional maturation of granulosa cells. Insulin signalling also stimulates mTOR complex 1. Phosphorylation of S6K and 4EBP1 by mTORC1 facilitates cellular growth and protein synthesis [78]. Excessive mTORC1 activation might disrupt the body’s metabolism. Chronic hyperinsulinemia exacerbates this risk in insulin-resistant states. Adiponectin interacts with insulin signalling at many levels. Activation of AdipoR1 and AdipoR2 induces AMPK phosphorylation. AMPK antagonises anabolic signalling induced by insulin. AMPK inhibits mTORC1 via phosphorylating TSC2 and raptor. Reducing mTORC1 activation inhibits excessive protein synthesis under low energy levels. Activation of AMPK enhances the body’s sensitivity to insulin. Enhanced insulin sensitivity reduces the necessary level of insulin. Reduced insulin exposure diminishes oxidative damage and atypical signalling pressure [79].

Adiponectin receptors reduce intracellular ceramide via their intrinsic ceramidase activity. The accumulation of ceramide impairs Akt signalling and induces cellular apoptosis. Reducing ceramide levels enhances the efficacy of insulin receptor signalling. The synthesis of sphingosine-1-phosphate facilitates survival signalling and maintains mitochondrial stability [80]. The use of glucose in the organism links insulin signals to oocyte sustenance. Increased glucose uptake by granulosa cells accelerates glycolysis. The synthesis of pyruvate and lactate increases. Monocarboxylate transporters and gap junctions facilitate the transfer of metabolites to the oocyte. Pyruvate energises the mitochondria in the oocyte for respiration [81]. Consequently, ATP synthesis increases. Sufficient ATP promotes spindle formation and chromosomal separation. Energy sufficiency enhances calcium oscillation capability during fertilisation. Calcium signalling requires ion pumps that use ATP and a consistent mitochondrial buffering capacity. Insulin resistance inhibits this sequence of occurrences. Reduced glucose absorption results in diminished availability of pyruvate. An insufficient substrate supply diminishes the functionality of oocyte mitochondria [82].

Steroidogenesis integrates metabolic signals with the regulation of the reproductive endocrine system. Adenylate cyclase is induced by the stimulation of FSH receptors. The concentrations of cyclic AMP increase, activating protein kinase A. PKA phosphorylates CREB and analogous transcription factors. Activation of CREB results in an increase in the levels of steroidogenic regulators [83]. StAR expression increases, enhancing the efficiency of cholesterol transport to mitochondria. CYP11A1 converts cholesterol into pregnenolone. Progesterone production transpires via a series of enzyme processes. Aromatase converts androgens into oestradiol. Regulating CYP19A1 is crucial for managing the production of oestradiol [67].

Insulin signalling alters the response of steroidogenic cells to FSH. Akt signalling may augment steroidogenic gene expression via affecting transcriptional regulators. In some ovarian conditions, insulin enhances IGF-1 signalling. The activation of the IGF-1 receptor and the signalling of the insulin receptor are identical processes. The activation of PI3K/Akt intensifies [84]. The simultaneous action of FSH and insulin signals may enhance the expression of steroidogenic enzymes. Hyperinsulinemia may potentially disrupt this equilibrium. Excess insulin may indirectly activate theca cells, perhaps resulting in increased androgen synthesis. The functionality of aromatase in granulosa cells may not exhibit a linear rise. Oestradiol synthesis may become dysregulated. Follicular maturation may be affected.

Adiponectin modifies steroidogenesis via AMPK and related mechanisms. The activation of AMPK may influence the production of aromatase. The direction of the impact is contingent upon the cellular context and the endocrine environment. During metabolic stress, AMPK may diminish the body’s steroidogenic drive. AMPK enhances mitochondrial function. Mitochondrial activity is essential for steroidogenesis, since the conversion of cholesterol begins inside mitochondria. The functionality of CYP11A1 is influenced by the integrity of the mitochondrial membrane. For StAR to transport cholesterol, the mitochondrial membranes must function correctly. Enhanced mitochondrial stability regulates steroid levels effectively [10].

Granulosa cells exhibit interaction between AMPK and cAMP signalling pathways. FSH stimulates the production of cAMP. cAMP activates PKA and subsequent transcriptional processes. Throughout this process, AMPK activation alters the cellular energy level. AMPK may alter the availability of coactivators for steroidogenic transcription. PGC-1α interacts with transcription factors and nuclear receptors. This integration influences steroidogenic gene networks. Excessive anabolic signalling without enough energy supply induces cellular stress. Activation of AMPK mitigates this discrepancy. Granulosa cells subsequently preserve viability and endocrine function [85].

This network is influenced by inflammatory signalling. Metabolic dysfunction increases low-grade inflammation. Granulosa cells may activate NF-κB in response to stress. Inflammatory signalling disrupts insulin receptor signalling by phosphorylating IRS proteins at serine residues. Under these circumstances, Akt activity diminishes. Adiponectin has anti-inflammatory properties across several tissues. Reducing the inflammatory tone may facilitate ovarian cells in transmitting signals to insulin. Enhanced insulin signalling improves glucose metabolism and the well-being of oocytes [86].

Adiponectin functions as a metabolic regulator that maintains stability inside the follicle. Enhanced AMPK activation facilitates mitochondrial function and promotes lipid catabolism. Reduced ceramide levels enhance Akt signalling and pathways that promote cellular survival [44]. Equilibrated mTOR activity facilitates optimal cellular function while minimising stress on the cells. Improved glucose metabolism facilitates the transport of pyruvate to the oocyte. Coordinated steroidogenesis promotes follicular maturation. Disruption of this integrated network may result in reduced oocyte competence and worse IVF results [87]. Mechanistic evidence supporting the direct role of adipokines and myokines in ovarian steroidogenesis and granulosa cell function is summarised in Table 2.

## 4. Irisin and Ovarian Metabolic Regulation

### 4.1. Irisin Synthesis, Receptor Signalling, and Downstream Pathways in the Ovary

Irisin is a peptide hormone generated by the proteolytic cleavage of fibronectin type III domain-containing protein 5. FNDC5 is a type I transmembrane glycoprotein located in skeletal muscle, adipose tissue, and some metabolically active organs [92]. The extracellular domain undergoes proteolytic degradation and is subsequently released as circulating irisin. Transcriptional coactivators involved in mitochondrial metabolism regulate the expression of the FNDC5 gene. The primary upstream regulator is peroxisome proliferator-activated receptor gamma coactivator 1 alpha. When cells need more energy, the expression of PGC-1α elevates [93]. Upon activation, AMPK phosphorylates transcription factors that are downstream of PGC-1α, hence facilitating PGC-1α transcription. Calcium-dependent signalling pathways may potentially facilitate the activation of PGC-1α. Increased activity of PGC-1α results in enhanced transcription of FNDC5. Increased FNDC5 expression results in increased irisin release into the bloodstream [94]. The circulating irisin reaches the ovary via the body’s circulatory system. Passive diffusion across the follicular vasculature enables ingress into follicular fluid. FNDC5 transcripts have been identified as being expressed locally in granulosa cells. This substantiates the notion that irisin might be synthesised inside the ovaries. Autocrine and paracrine actions may therefore augment endocrine signalling [95].

Irisin has been detected in follicular fluid collected during controlled ovarian stimulation. Concentrations vary among follicles and may signify localised metabolic activity. Under certain metabolic circumstances, granulosa cells synthesise FNDC5 mRNA and protein [90]. This indicates regional regulation of irisin production inside the follicle. The first phase of cellular response to irisin occurs when it attaches to receptors on the plasma membrane. Integrin receptor complexes including αV subunits have been identified as functional binding sites. Integrins function as transmembrane receptors that link external impulses to intracellular pathways. Upon ligand binding, an integrin undergoes a conformational shift [96]. Upon integrin interaction, focal adhesion kinase is activated. The phosphorylation of FAK initiates signalling cascades inside cells. Upon activation of receptor complexes, Src family kinases are recruited. This method initiates the MAPK and PI3K signalling pathways. Signal transmission induces alterations in transcription regulation and the synthesis of metabolic enzymes [97].

A primary impact of irisin signalling inside cells is the activation of AMPK. AMPK functions as a cellular energy sensor that responds to fluctuations in AMP and ATP concentrations. Exposure to Irisin increases the phosphorylation of AMPK at regulatory regions. When AMPK is activated, cells initiate pathways that generate ATP [98]. The phosphorylation of acetyl-CoA carboxylase enhances fatty acid oxidation. Reducing malonyl-CoA concentrations inhibits the activity of carnitine palmitoyltransferase-1. CPT1 facilitates the transport of fatty acids into mitochondria. An increased influx of fatty acids into the cell accelerates β-oxidation. Augmented lipid metabolism results in increased acetyl-CoA synthesis [99]. An increased concentration of acetyl-CoA enhances the efficiency of the tricarboxylic acid cycle. An increased production of NADH and FADH_2_ accelerates the electron transport cycle. When these events occur, ATP synthesis is enhanced. Increased ATP availability enhances the metabolic functions of granulosa cells. Enhanced metabolic efficiency increases the availability of substrates for the oocyte [100].

Irisin signalling facilitates the production of more mitochondria via regulating transcription. Activation of AMPK enhances the production of PGC-1α, a transcriptional coactivator that regulates nuclear genes encoding mitochondrial proteins. Nuclear respiratory factors NRF1 and NRF2 regulate the synthesis of respiratory chain subunits [101]. Mitochondrial transcription factor A regulates the replication and transcription of mitochondrial DNA. Increased TFAM expression results in a greater quantity of mitochondrial DNA copies. An increased quantity of mitochondrial DNA enhances mitochondrial efficiency during respiration. An enhanced quantity of respiratory chain proteins enhances the efficiency of oxidative phosphorylation. Enhanced mitochondrial activity results in increased ATP production. An increased availability of ATP aids cells in biosynthetic and regulatory functions. An improved number of mitochondria enhances a cell’s capacity to manage metabolic stress. Mitochondrial biogenesis maintains metabolic stability throughout time [102].

The mechanisms of fusion and fission regulate the structural integrity of mitochondria. Irisin signalling influences the expression of proteins that regulate mitochondrial movement. Mitofusin-1 and mitofusin-2 regulate the fusing of the outer mitochondrial membrane. Fusion enables the interchange of mitochondrial proteins and mitochondrial DNA. Functional complementation among mitochondria improves total mitochondrial efficiency [103]. Optic atrophy protein 1 regulates the fusing of the inner mitochondrial membrane. Adequate fusion maintains the stability of the mitochondrial membrane potential. Dynamin-related protein 1 regulates mitochondrial fission. Mitophagy is a mechanism by which fission eliminates defective components of mitochondria. Equilibrium between fusion and fission maintains mitochondrial integrity. Irisin signalling maintains the integrity and functionality of mitochondria [104].

The generation of reactive oxygen species is intricately linked to mitochondrial respiration. Superoxide radicals are generated when electrons escape from respiratory complexes. Superoxide dismutase converts superoxide into hydrogen peroxide. In the absence of neutralisation, hydrogen peroxide may generate hydroxyl radicals. Irisin signalling enhances the synthesis of antioxidant enzymes [105]. An increased presence of superoxide dismutase, catalase, and glutathione peroxidase in cells enhances their capacity to combat free radicals. Reduced oxidative stress preserves mitochondrial DNA integrity. Reduced oxidative damage enhances the efficiency of electron transport. Maintaining mitochondrial structural integrity is essential for sustaining ATP synthesis. An improved redox balance enhances the viability of granulosa cells [106].

The viability of granulosa cells depends on the modulation of apoptotic signalling pathways. Irisin signalling activates the PI3K and Akt pathways. Phosphorylation of Akt promotes signalling that inhibits apoptosis. Activation of Akt results in an increase in Bcl-2 levels. Anti-apoptotic proteins preserve the integrity of the mitochondrial membrane [107]. Under these circumstances, Bax activity diminishes. A diminished activity of Bax impedes the permeability of the outer mitochondrial membrane. The release of cytochrome c into the cytoplasm seems improbable. Reduced cytochrome c release inhibits caspase activation. Reducing caspase activation preserves cellular viability. Maintaining the viability of granulosa cells promotes follicular development [108].

Irisin signalling also influences the metabolism of glucose. Activation of AMPK enhances the expression of glucose transporters. Elevated concentrations of GLUT1 and GLUT4 facilitate glucose uptake by granulosa cells. An increased concentration of glucose inside cells enhances the efficiency of glycolysis. Pyruvate and lactate are produced during glycolysis. Pyruvate enters the mitochondria and facilitates oxidative phosphorylation. Increased respiration in the mitochondria results in enhanced ATP production. An improved availability of ATP facilitates the synthesis of new molecules by cells. Granulosa cells provide the oocyte with essential nutrients for its development. Enhanced substrate availability facilitates mitochondrial metabolism in oocytes. Enhanced metabolic support for oocytes improves their developmental capacity [109].

Mitochondrial activity and metabolic control are crucial for steroidogenic function. The first stage in steroid hormone synthesis involves the translocation of cholesterol into mitochondria. The relocation of substances is regulated by steroidogenic acute regulatory protein. Irisin signalling may influence the expression of steroidogenic enzymes. Enhanced mitochondrial activity increases pregnenolone synthesis. An increase in pregnenolone facilitates the body’s production of oestradiol and progesterone. The synthesis of steroid hormones regulates follicular development. Effective steroidogenesis facilitates oocyte maturation [110].

Irisin signalling also interacts with insulin signalling pathways. Activation of AMPK enhances the sensitivity of insulin receptors. Enhanced insulin signalling facilitates glucose uptake by cells. Enhanced glucose metabolism improves mitochondrial function. Reduced metabolic stress enhances cellular viability. This metabolic control improves the functionality of granulosa cells. Enhanced functionality of granulosa cells provides superior metabolic support to oocytes [111].

Irisin concentrations in the follicle may indicate mitochondrial functionality and metabolic health. Reduced irisin signalling may impede mitochondrial formation. Reduced mitochondrial efficacy results in diminished ATP synthesis. Reduced ATP availability impedes the process of meiosis. The functionality of an oocyte is contingent upon the efficiency of its mitochondria. Irisin is a crucial regulator of follicular metabolism and reproductive function.

### 4.2. Irisin and Mitochondrial Bioenergetics in Granulosa Cells and the Oocyte

Mitochondrial bioenergetics is essential to the functionality of granulosa cells and the efficacy of oocyte performance. The electron transport chain must function well for ATP synthesis to occur. In the tricarboxylic acid cycle, NADH and FADH_2_ transfer electrons to respiratory complexes. Complex I transfers electrons from NADH to coenzyme Q. Complex II incorporates electrons derived from the catabolism of succinate [112]. The electron transport continues via complex III and cytochrome c. Complex IV transfers electrons to molecular oxygen, resulting in the formation of water. An electrochemical gradient is established when protons traverse the inner mitochondrial membrane. ATP synthase converts this gradient into ATP. Proper ATP synthesis is essential for cytoskeletal structure and intercellular signalling. Ion transport and protein phosphorylation are two instances of energy-dependent activities reliant on ATP synthesis in mitochondria. Oocyte meiotic development requires sustained ATP availability. ATP-dependent microtubule dynamics facilitate spindle assembly and chromosomal alignment [113].

Irisin enhances the bioenergetic capability of mitochondria via altering gene expression and protein synthesis. The activation of AMPK is a crucial preliminary step. AMPK phosphorylation elevates the levels of PGC-1α. PGC-1α regulates the transcription of nuclear genes encoding mitochondrial respiratory proteins. Increased expression of the respiratory chain subunits enhances the efficiency of electron transport [114]. Enhanced electron flow reduces electron leakage and oxidative stress. Mitochondrial transcription factor A regulates the replication of the mitochondrial genome. Increased TFAM activity results in a greater quantity of mitochondrial DNA copies. An increased quantity of mitochondrial DNA facilitates the synthesis of components of the respiratory chain encoded by mitochondrial genes. An increased number of mitochondria correlates with enhanced overall respiratory capacity. An increased number of mitochondria enhances a cell’s capacity to manage metabolic stress [115].

Intact mitochondrial DNA is essential for optimal oxidative phosphorylation. Mitochondrial DNA encodes the directives for synthesising essential components of respiratory chain complexes. Oxidative damage to mitochondrial DNA impairs respiratory function. Irisin signalling enhances the body’s protection against free radicals [116]. An increase in superoxide dismutase results in a reduction in superoxide accumulation. Glutathione peroxidase converts hydrogen peroxide into non-toxic compounds. Reduced oxidative stress prevents damage to mitochondrial DNA. Maintaining the integrity of mitochondrial DNA ensures optimal function of the respiratory chain. Enhanced respiration facilitates the continuous synthesis of ATP [117]. Enhanced mitochondrial stability aids granulosa cells in performing their metabolic functions.

Granulosa cells provide the metabolic substrates necessary for oocyte mitochondrial respiration. Pyruvate produced during glycolysis enters the oocyte via connexin channels and monocarboxylate transporters. In oocyte mitochondria, pyruvate is converted into acetyl-CoA. Acetyl-CoA enters the tricarboxylic acid cycle, facilitating the production of NADH [23]. The electron transport chain operates more efficiently when substrate availability is sufficient. Irisin signalling enhances the glycolytic activity of granulosa cells. Increased glucose consumption results in elevated pyruvate production. Enhanced availability of substrates improves oocyte mitochondrial respiration. Increased ATP synthesis facilitates oocyte maturation. Enhanced mitochondrial function accelerates cytoplasmic maturation.

The mitochondrial membrane potential indicates the efficiency of mitochondrial function. Proton gradients across the inner mitochondrial membrane maintain the membrane potential. A decrease in membrane potential indicates dysfunction in the mitochondria. A diminished membrane potential impedes ATP synthesis [118]. Signalling by Irisin preserves the integrity of the mitochondrial membrane. An increased production of mitochondrial fusion proteins maintains the integrity of mitochondrial structure. Mitofusin proteins facilitate the fusion of mitochondrial membranes. Fusion enables the interchange of mitochondrial components. Functional complementation among mitochondria improves respiratory efficiency. Enhanced mitochondrial architecture results in increased ATP synthesis [119].

Mitochondrial quality control mechanisms eliminate defective mitochondria. Mitophagy eliminates dysfunctional components of mitochondria. Irisin signals may influence mitophagy via the activation of AMPK. AMPK regulates proteins associated with autophagy that participate in mitochondrial turnover [120]. Eliminating damaged mitochondria preserves the integrity of the mitochondrial population. An enhanced quality of the mitochondrial population augments the metabolic efficiency of cells. Enhanced mitochondrial activity promotes the survival of granulosa cells. Preserving the metabolic function of granulosa cells facilitates oocyte development [121].

The presence of ATP directly influences meiotic competence. ATP-dependent mechanisms are essential for microtubule polymerisation. The stability of the spindle is contingent upon the availability of energy. The cytoskeleton must function adequately for chromosomes to align properly. Adequate ATP is essential for the formation of spindles; insufficient levels hinder this process. Errors in chromosomal segregation may occur due to insufficient energy. The stimulation of mitochondria by irisin enhances ATP generation. Enhanced availability of ATP facilitates the efficient progression of meiosis. More stable chromosomes facilitate the process of fertilisation [122].

Mitochondrial activity is crucial for calcium transmission during fertilisation. Mitochondria regulate intracellular calcium storage. Following fertilisation, calcium oscillations activate developmental pathways. ATP-dependent calcium pumps regulate intracellular calcium levels. When mitochondrial function is impaired, calcium regulation is also compromised [121]. Irisin signalling enhances mitochondrial function and increases ATP production. Enhanced calcium control facilitates the usual process of fertilisation. Enhanced mitochondrial function augments the potential for embryonic development. Clinical investigations have shown associations between follicular fluid irisin levels and ovarian response. Individuals who have poor responses often demonstrate alterations in their metabolic signalling. Reduced irisin signalling may impede mitochondrial growth. Reduced mitochondrial activity results in diminished ATP production. Compromised ATP synthesis affects oocyte viability. Improved mitochondrial activity may clarify the relationships between irisin and reproductive outcomes. Irisin may indicate the activity level of mitochondria inside the follicle [123].

Irisin enhances mitochondrial function by simultaneously regulating mitochondrial biogenesis, antioxidant defence, and metabolic signalling. Increased mitochondrial activity improves the energy utilisation of granulosa cells. Improved metabolism in granulosa cells facilitates the metabolic processes of the oocyte. Enhanced mitochondrial function increases oocyte competence. Irisin influences reproductive function primarily via mitochondrial control. The majority of the knowledge on irisin is derived from limited cohorts or experimental studies. Information about human IVF remains limited, and the correlations identified between ovarian response and embryonic development are not consistently seen across all research. The molecular mechanisms through which adiponectin, irisin, and asprosin regulate follicular metabolism and oocyte competence are summarised in Table 3.

## 5. Asprosin and Emerging Metabolic Regulation in the Ovary

Asprosin is a glucogenic peptide hormone derived from the degradation of profibrillin, which is produced by the FBN1 gene. Fibrillin-1 is a structural glycoprotein located in the microfibrils of the extracellular matrix. The proteolytic cleavage of the C-terminal portion of profibrillin generates circulating asprosin. This cleavage occurs post-translation in secretory tissues [124]. Adipose tissue is the primary reservoir of asprosin in the bloodstream. Plasma concentrations increase with fasting or metabolic stress. Increased asprosin secretion aids in maintaining constant glucose levels throughout the body. Asprosin traverses the bloodstream to reach peripheral organs. Entry into ovarian follicular fluid likely occurs by passive diffusion via the blood arteries around the follicle. Recent experimental discoveries demonstrate the existence of asprosin in reproductive tissues [125].

The preponderance of current research about asprosin signalling originates from metabolic organs, such as the liver and adipose tissue, whereas direct data in the ovarian environment remains limited. Asprosin has been shown to stimulate G protein-coupled receptor pathways, leading to increased adenylate cyclase activity and the consequent activation of the cAMP-PKA signalling cascade [126]. This route regulates critical metabolic activities, including glucose release and energy balance. However, it is yet unclear if asprosin triggers the same signalling pathways in ovarian cells [127] Although cAMP–PKA signalling is well described in granulosa cells, particularly regarding steroidogenesis, direct data linking asprosin to this route in the follicular milieu is lacking. Furthermore, the expression and functional importance of asprosin receptors in ovarian tissue are little defined, consequently limiting mechanistic understanding [128].

Cyclic AMP signalling is a primary regulator of ovarian function, with protein kinase A regulating the transcription of essential enzymes involved in steroidogenesis. The expression of StAR and CYP11A1 is modulated by cAMP-dependent pathways, promoting cholesterol transport into mitochondria and the consequent production of pregnenolone [127]. Nevertheless, the majority of molecular findings connecting cAMP signalling to asprosin function derive from research conducted in metabolic tissues rather than the ovarian context. Although the roles of cAMP and steroidogenic signalling in granulosa cells are well documented, direct evidence for asprosin’s regulatory function in the follicular milieu is still few [129]. Asprosin is suggested to affect metabolic pathways, including AMPK and insulin signalling, mostly based on research from non-ovarian systems. In this context, the possible impacts on mitochondrial function and energy balance in granulosa cells should be regarded with care, since tissue-specific variations in receptor expression and intracellular signalling mechanisms may considerably alter these responses [130].

Asprosin influences insulin signalling pathways. The activation of the insulin receptor regulates glucose uptake and bodily functions. Asprosin has been proposed to regulate the release of glucose into the body and the sensitivity of insulin. Elevated blood glucose levels alter the quantity of insulin released [125]. Alterations in insulin signalling impact the metabolic function of granulosa cells. Modifications in PI3K and Akt signalling is involved in the functionality of glucose transporters. Reduced glucose absorption inhibits glycolytic flux. Reduced glycolytic flux leads to decreased pyruvate availability, thereby impairing mitochondrial oxidative phosphorylation. Mitochondrial function depends on adequate substrate availability and regulatory signalling. Asprosin-mediated metabolic signalling regulates mitochondrial efficiency. Variations in substrate availability influence the functionality of the tricarboxylic acid cycle. Reduced substrate availability complicates the synthesis of NADH and FADH_2_. Decreased activity in the electron transport chain results in less ATP production. Reduced ATP availability alters cellular synthesis processes. The metabolism of granulosa cells may be influenced under various circumstances [131].

Asprosin signals contribute to the regulation of oxidative stress. Alterations in glucose catabolism accelerate the influx of substrates into the mitochondria. Increased electron transport activity results in the production of a greater quantity of reactive oxygen species. Elevated concentrations of reactive oxygen species impair mitochondrial function [105]. Oxidative damage impacts mitochondrial DNA and proteins throughout the respiratory chain. Less efficient mitochondria impede ATP production. Oxidative stress induce cellular metabolic instability. Metabolic stress influences apoptotic signalling pathways. Mitochondrial dysfunction activates pathways that induce apoptosis. The release of cytochrome c initiates caspase-dependent apoptosis [34]. The viability of granulosa cells depends on mitochondrial integrity. Asprosin-induced alterations in metabolism influence apoptotic signals. Reduced metabolic stability increases susceptibility to apoptosis. The depletion of granulosa cells impedes follicular growth. Asprosin signalling may influence the regulation of metabolic gene expression. PKA activation regulates transcription factors such as CREB. The phosphorylation of CREB alters the expression of genes associated with metabolism and steroidogenesis. Alterations in gene expression influence cellular energy use. Alterations in transcriptional regulation impact mitochondrial biogenesis and energy metabolism [132].

Variations in asprosin concentrations are associated with metabolic diseases. Insulin resistance and obesity are associated with elevated levels of asprosin. These disorders are associated with impaired ovarian metabolic function. Alterations in metabolic signalling may diminish the efficacy of granulosa cells. Reduced mitochondrial efficiency may impair the functional capacity of oocytes [133]. Asprosin indicates a link between systemic metabolic malfunction and reproductive disability. Asprosin is a novel metabolic regulator that plays a significant role in ovarian function. Its signalling pathways influence glucose utilisation, mitochondrial function, and steroidogenesis. The regulation of cellular energy balance affects granulosa cell activity and follicular growth. Further investigation is required to elucidate the impact of asprosin on assisted reproduction and the functionality of oocytes [134]. Due to the scarcity of direct ovarian data, extrapolation from other metabolic organs should be approached cautiously, particularly given potential variations in receptor expression and intracellular signalling contexts.

Equilibrium in metabolic signalling is crucial for mitochondrial functionality inside the follicle. Asprosin influences the body’s capacity to maintain steady glucose levels. Alterations in glucose dynamics affect substrate availability inside cells. Elevated blood glucose levels increase glycolytic flow in granulosa cells. Improved concentrations of glycolytic intermediates facilitate substrate entry into the mitochondria. Increased acetyl-CoA synthesis enhances the efficiency of the tricarboxylic acid cycle. Elevated NADH synthesis accelerates the progression of the electron transport cycle [135]. An increased flow of electrons results in the generation of greater proton gradients. Excessive electron flow may result in increased electron leakage at complexes I and III. Under these circumstances, the production of superoxide increases. Elevated concentrations of superoxide induce oxidative stress [136].

The accumulation of reactive oxygen species damages the mitochondria. Superoxide damages iron–sulphur clusters inside respiratory complexes. Mitochondrial lipids are susceptible to oxidation by hydrogen peroxide. The oxidation of cardiolipin diminishes the stability of the inner mitochondrial membrane [137]. Compromise of the membrane’s integrity impedes the maintenance of the proton gradient. When the membrane potential decreases, ATP synthase exhibits less efficacy. Under mitochondrial stress, ATP synthesis diminishes. Adequate ATP production is essential for granulosa cells to perform their metabolic functions effectively. Under low-energy circumstances, oocyte support is diminished [35].

Oxidative stress impacts mitochondrial DNA. Mitochondrial DNA lacks protective histone structures. Oxidative damage to mitochondrial DNA impedes the transcription of respiratory chain constituents. Reduced expression of mitochondrial-encoded proteins diminishes respiratory efficiency. Reduced oxidative phosphorylation results in diminished ATP synthesis. Chronic oxidative stress induces mitochondrial permeability change. The activation of the permeability transition pore alters the membrane potential. The release of cytochrome c initiates apoptotic pathways reliant on caspases. The apoptosis of granulosa cells compromises the integrity of the follicle [138].

Insulin resistance increases metabolic stress inside the follicle. In situations of insulin resistance, asprosin levels are often elevated above the normal range. Insulin resistance alters the functionality of PI3K/Akt signalling. Reduced Akt phosphorylation impedes the translocation of glucose transporters. Impaired glucose absorption reduces controlled glycolytic regulation [139]. Fluctuating glucose concentrations inside cells destabilise mitochondrial metabolism. Impaired insulin signalling also increases the phosphorylation of serine in proteins that serve as substrates for insulin receptors. The altered IRS function diminishes downstream metabolic signalling. Reduced metabolic flexibility increases the susceptibility of cells to oxidative damage [140].

Prolonged metabolic overload may deactivate AMPK signalling. Reduced AMPK activation results in diminished fatty acid oxidation. The accumulation of lipid intermediates increases lipotoxic stress. The accumulation of ceramide and diacylglycerol impairs mitochondrial activity. Lipid-induced mitochondrial dysfunction increases the production of reactive oxygen species. Reduced fatty acid oxidation impedes the ability of lipid substrates to generate ATP. Metabolic inflexibility obstructs cellular response to energy demands. Under these circumstances, oocyte metabolic support diminishes [141].

Operational mitochondrial function is essential for steroidogenesis. The transfer of cholesterol into mitochondria is the most significant rate-limiting step in steroid production. The trafficking is facilitated by steroidogenic acute regulatory protein. Disruption of the mitochondrial membrane impedes StAR functionality. Reducing cholesterol transfer inhibits the synthesis of pregnenolone. The activity of CYP11A1 depends on the integrity of mitochondrial membranes. Reduced pregnenolone synthesis impedes the generation of progesterone and oestradiol. Oxidative stress appears to reduce aromatase activity. Reduced oestradiol levels inhibit granulosa cell proliferation and follicular maturation [110].

Oxidative stress alters the transcriptional regulation of steroidogenic enzymes. Stress-responsive mechanisms such as NF-κB may inhibit the production of steroidogenic genes. Inflammatory signalling interferes with normal endocrine signalling. Altered cytokine profiles influence the development of granulosa cells [48]. Chronic oxidative stress diminishes the body’s responsiveness to follicle-stimulating hormone. Reduced cAMP levels inhibit PKA from triggering the transcription of steroidogenic genes. Hormonal imbalance affects follicular development and oocyte maturation.

Malfunctioning granulosa cells in mitochondrial function directly impact oocyte competence. Adequate ATP is essential for the efficient transport of pyruvate and the functionality of metabolic support mechanisms. Reduced antioxidant levels increase the susceptibility of the oocyte to oxidative damage. Oxidative stress damages spindle microtubules and disrupts chromosome alignment [142]. Deficient mitochondrial inheritance affects early embryonic development. Alterations in the metabolic milieu inside the follicle may diminish the rates of fertilisation and blastocyst development.

Clinical findings link metabolic problems to impaired reproductive outcomes. Elevated asprosin levels in individuals with insulin resistance indicate metabolic distress. Similar metabolic disturbances impact the follicular compartment. Impaired mitochondrial activity and oxidative imbalance contribute to reduced IVF success rates. Asprosin appears to serve as a biochemical marker signifying follicular bioenergetic stress [143]. Asprosin-induced alterations in metabolism are thought to influence mitochondrial efficiency, redox reaction stability, and steroidogenesis capability. Interruption of these mechanisms compromises follicular function. The efficacy of oocytes depends on the preservation of mitochondrial integrity and metabolic balance. Further investigation is required to determine whether asprosin directly affects ovarian bioenergetics or indicates a broader systemic metabolic disorder.

## 6. Integrated Molecular Crosstalk Between Adiponectin, Irisin, Asprosin, and Follicular Bioenergetics

Adipokines and myokines do not function via distinct routes; instead, they intersect at a common set of metabolic activities inside the follicular milieu. This integration is crucial since mitochondrial function, redox equilibrium, and steroidogenic activity together influence oocyte performance. Irisin, asprosin, and adiponectin are signals that interact with energy-sensing pathways like as AMPK, AKT, and mTOR. This establishes a regulatory network that adapts according to the organism’s metabolic state [144].

Such pathways do not work on their own. Activation of AMPK may modify mTOR signalling, influencing cellular growth and protein synthesis, while its interaction with AKT pathways integrates metabolic and hormonal signals. Mitochondrial function and redox control collaborate to establish a feedback loop. Elevated oxidative stress levels may impair signalling efficacy and diminish steroidogenic ability. This linked network suggests that metabolic signals are integrated rather than conveyed in a linear manner within the follicular environment [145,146].Through its interaction with PGC-1α, AMPK links energy sensing to mitochondrial function, reinforcing the dependence of oocyte competence on metabolic balances [147].

Insulin and growth factor signals converge in the PI3K/Akt signalling pathway. Insulin binding to its receptor results in the addition of a phosphate group to IRS proteins. PIP3 is synthesised from PIP2 by PI3K, subsequently activating PDK1 and Akt. Akt facilitates cellular glucose uptake and its subsequent progression via glycolysis. An increase in glycolysis results in a greater availability of pyruvate. Pyruvate facilitates mitochondrial respiration in granulosa cells and the oocyte. Akt signalling furthermore promotes cell survival by regulating proteins within the Bcl-2 family. Inhibition of FOXO1 enhances cellular proliferation and functional differentiation. Akt activation often precedes mTORC1 activation. Robust Akt-mTOR signalling accelerates protein synthesis. Overactivation may be harmful in the setting of insulin resistance [148].

Adiponectin enhances insulin signalling via many methods. Activation of AMPK improves metabolic flexibility and reduces oxidative stress. Adiponectin receptors reduce intracellular ceramide levels via the action of ceramidase. Ceramide disrupts Akt signalling and promotes apoptosis [149]. Reducing ceramide levels enhances the efficacy of the insulin signalling system. The synthesis of sphingosine-1-phosphate facilitates survival signalling. Enhanced insulin sensitivity reduces the need for insulin. Reduced insulin exposure inhibits the continuous activation of mTORC1. Consequently, granulosa cells have a more consistent metabolic profile [150].

Irisin facilitates optimal mitochondrial function and maintains redox equilibrium. Activating AMPK and PGC-1α enhances mitochondrial proliferation and functionality. Enhanced electron transport results in less electron leakage. Reduced leakage results in less superoxide production. Irisin signalling enhances the activity of antioxidant enzymes. The activity of superoxide dismutase and glutathione peroxidase increases. Glutathione recycling maintains the redox equilibrium inside cells. Enhanced redox regulation reduces the likelihood of oxidative stress-induced damage to mitochondrial DNA. The mitochondrial membrane potential remains more constant. ATP synthesis becomes more reliable throughout the energy-demanding phases of follicular development.

Asprosin may influence follicular metabolism via cAMP-dependent mechanisms associated with insulin. cAMP and PKA signalling influence the control of ovarian steroidogenesis. cAMP enhances StAR expression and facilitates cholesterol transport into mitochondria. Excessive metabolic substrate flow may lead to increased production of ROS by mitochondria [125]. Insulin resistance often coexists with elevated asprosin levels. Impaired Akt signalling reduces regulated glucose absorption. When cells exhibit insulin resistance, their energy equilibrium becomes destabilised. The activation of AMPK is thought to be insufficient to compensate for metabolic overload. Lipid intermediates accumulate, exerting additional stress on lipotoxic cells. Mitochondrial fragmentation may occur with extended stress. Fission induced by DRP1 may increase, although fusion capability decreases. When mitochondria malfunction, they produce more ROS and cease steroidogenesis [151].

Redox signalling provides an additional dimension of integration. NRF2 regulates the expression of antioxidant genes by detecting them via KEAP1. Oxidative stress releases NRF2 from KEAP1. NRF2 translocates to the nucleus and activates antioxidant response elements. Elevating antioxidant enzyme levels facilitates the neutralisation of ROS [152]. Activation of AMPK enhances the efficacy of NRF2 by regulating energy levels and reducing oxidative stress. Adiponectin and irisin indirectly augment NRF2-mediated antioxidant responses. Chronic metabolic excess may surpass NRF2’s capabilities. Oxidative damage accumulates when antioxidant mechanisms are unable to maintain equilibrium. Injury to mitochondrial DNA and oxidation of cardiolipin impair the functionality of the respiratory chain [153].

Several testable hypotheses are generated by this integrated framework. Initially, differences in the patterns of adipokines and myokines in follicular fluid may indicate different metabolic states that directly affect mitochondrial efficiency and oocyte competence. Secondly, the modification of AMPK-mTOR signalling balance may serve as a possible target for enhancing oocyte quality. The integrated evaluation of metabolic and redox indicators in follicular fluid may enhance the prediction of IVF results beyond traditional measures [154].

Clinically speaking, this integrated metabolic model might help explain the variation in ovarian response, embryo growth, and implantation results seen throughout IVF cycles. A network-based strategy that examines metabolic, mitochondrial, and redox state, rather than relying on a single biomarker, may provide more precise predictive information and facilitate the development of tailored therapies [154].

**Figure 1:**
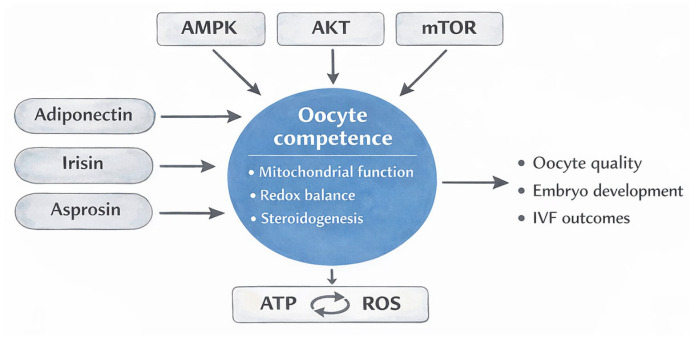
Integrated metabolic signalling in the follicular microenvironment and its impact on oocyte competence.

A schematic illustrating the interaction between adipokines and myokines inside the follicular environment, in Figure 1. Adiponectin, irisin, and asprosin are signals that converge on critical metabolic pathways such as AMPK, AKT, and mTOR. These pathways influence mitochondrial function, redox equilibrium, and steroidogenesis in granulosa cells and the oocyte. These interconnected pathways improve oocyte competency and ultimately appear in embryo development and IVF outcomes.

## 7. Discussion

This narrative review underscores that metabolic hormones originating from adipose tissue and skeletal muscle function not only as systemic indicators of energy balance but also as active regulators within the follicular microenvironment, influencing granulosa cell metabolism, steroidogenesis, and ultimately oocyte competence. Chang et al. provided initial clinical evidence suggesting that adiponectin concentrations within individual follicles are positively correlated with normal fertilisation rates, indicating that adiponectin reflects a metabolically favourable follicular environment rather than merely systemic metabolic status [13]. Qin et al. elaborated on these findings by demonstrating dynamic fluctuations between serum and follicular adiponectin compartments during IVF phases, emphasising that compartmentalised adiponectin regulation may influence reproductive success independent of absolute circulating concentrations [56]. Acet et al. and Bousmpoula et al. further validated the metabolic integration of the follicular environment by demonstrating the presence of irisin in follicular fluid, which is associated with insulin resistance, androgen levels, and ovarian response phenotypes [17,60]. This indicates that myokine signalling directly mirrors and may influence follicular metabolic homeostasis. Maylem et al. and Batalha et al. provided mechanistic evidence demonstrating that asprosin is expressed in ovarian somatic cells and directly regulates steroidogenesis and follicular endocrine activity, thereby establishing asprosin as a novel metabolic regulator of ovarian physiology [18,19]. In conclusion, these results endorse a model wherein adiponectin, irisin, and asprosin function as integrative metabolic signals linking systemic energy homeostasis with local mitochondrial activity, steroidogenic potential, and in vitro fertilisation outcomes.

Chang et al. proposed that follicular adiponectin is associated with fertilisation competence at the level of the single dominant follicle, as higher levels of follicular adiponectin correlated with normal fertilisation in non-obese women, with fertilisation rates increasing across adiponectin tertiles [13]. In contrast, leptin primarily indicated BMI and showed only non-significant trends concerning maturity and embryo quality. Qin et al. challenged the oversimplified notion that “higher adiponectin levels are invariably advantageous” by revealing no significant difference in absolute adiponectin concentrations between successful and failed pregnancies in serum or follicular fluid [56]. They detected a compartment signal showing that blood adiponectin levels exceeded follicular adiponectin levels on the day of oocyte extraction in failed cycles. Dafopoulos et al. validated the idea that timing and endocrine context are crucial, since serum adiponectin remained stable throughout stimulation but decreased in the luteal phase, indicating that adiponectin physiology during IVF is phase-dependent rather than static [57]. Takikawa et al. determined that intrafollicular insulin serves as a more substantial predictor of pregnancy compared to leptin or adiponectin, observing that heightened follicular insulin levels correlate with non-pregnant cycles and are especially pronounced in PCOS, thereby endorsing a model wherein metabolic overload can affect outcomes despite seemingly favourable adiponectin signals [58]. Athar et al. developed a system-level framework for these discrepancies, emphasising that metabolic hormones operate within the hypothalamic-pituitary-gonadal axis and the ovary, suggesting that follicular readouts represent a localised “integration” of central energy sensing, gonadotropin stimulation, and ovarian cell metabolism [155]. Zhang et al. validated this by defining follicular fluid as a composite biofluid that combines plasma transudate and granulosa secretion, clarifying the variable behaviour of single-analyte indicators across different aetiologies, methods, and outcome definitions [23].

Merhi et al. established the mechanistic validity of adiponectin as more than a simple biomarker by correlating follicular adiponectin concentrations with serum AMH levels and by demonstrating that adiponectin deficiency in mice altered ovarian transcripts in pathways relevant to follicular development, including kisspeptin signalling and AMH receptor expression [91]. Barbe et al. linked receptor biology to ovarian function by delineating the expression patterns of AdipoR1 and AdipoR2 across the reproductive axis and by elucidating the impact of adiponectin on steroidogenesis, proliferation, apoptosis, and oxidative stress in ovarian somatic cells [69]. This illustrates a biochemical pathway from the body’s metabolic condition to follicular competence. Estienne et al. reinforced this translation by presenting evidence that adipokines affect oocyte maturation and early embryo development via metabolic signalling rather than exclusively through endocrine pathways, indicating that adiponectin may influence competence without causing substantial, consistent changes in embryo morphological metrics [156]. Li et al. demonstrated that ovarian reserve phenotypes may affect adipokine interpretation, as follicles with diminished ovarian reserve displayed altered adipokine cytokine profiles, and embryo quality was more strongly correlated with IL-6 than with the adipokine panel, thereby supporting the notion that intrafollicular inflammation and stress signalling can outweigh adipokine effects [65]. Chang et al. thus accord optimum with a “conditional benefit” hypothesis, in which adiponectin yields insights just when inflammation, insulin resistance, and oxidative stress are not prominent in follicular physiology [13]. Qin et al. strengthened that conditional model by suggesting that the serum-to-follicular adiponectin gradient may signify compromised follicular uptake, altered binding complexes, or disturbed paracrine equilibrium, all of which might decouple circulating adiponectin from local granulosa-cumulus functionality [56].

Acet et al. shifted the discussion from adipokines to myokines by demonstrating the presence of irisin in both serum and follicular fluid during IVF and by revealing that compartment behaviour differs by phenotype, exhibiting a notable serum follicular gap in poor responders, unlike in PCOS [17]. Bousmpoula et al. similarly discovered that individuals with PCOS and those who are overweight have elevated levels of irisin [60]. They associated irisin with BMI and dyslipidemia. However, they were unable to establish a connection between follicular irisin and pregnancy. This indicates that irisin may serve as a superior indicator of metabolic condition compared to a direct clinical prediction. Poretsky et al. clarified a direct ovarian-cell mechanism connecting irisin to competence by showing that irisin upregulated CYP19A1 in primary human granulosa cells, indicating enhanced aromatase capacity and a possible modification in intrafollicular oestradiol synthesis under particular exposure conditions and dosages [88]. Stojchevski et al. enhanced the mechanistic framework by revealing an 84-gene qPCR array response in human granulosa cells after irisin exposure, thereby substantiating the hypothesis that irisin can reprogram a network of fertility-related transcripts rather than operating through a singular steroidogenic pathway [89]. Daudon et al. issued a critical caution concerning species and context by demonstrating in bovine granulosa cells that irisin reduced GLUT1/3/4 transcripts, increased lactate release, and suppressed FSH- and IGF-1-mediated estradiol and progesterone secretion via MAPK3/1, rather than through Akt or AMPK signalling, which contradicts the oversimplified narrative that “irisin is pro-steroidogenic [90]”. Poretsky et al. and Daudon et al. collectively assert that the effects of irisin depend on species, granulosa differentiation status, co-gonadotropin environment, and downstream pathway interactions, making the mixed clinical correlations in IVF cohorts biologically plausible rather than mere statistical artefacts [88,90]. Acet et al. and Bousmpoula et al. support the concept of conditionality by linking irisin to insulin resistance indicators, implying that irisin may serve as a compensatory metabolic response that is elevated in dysmetabolic situations while concurrently exhibiting reduced efficacy [17,60]. Takikawa et al. support this rationale by illustrating that insulin-related follicular stress may be paramount, requiring that any irisin signal be assessed within the framework of intrafollicular insulin levels, glycolytic activity, and subsequent oxidative burden [58].

Maylem et al. identified asprosin as a distinct fasting-associated adipokine axis in follicular biology by elucidating the developmental regulation of FBN1, FURIN, and the putative receptor OR4M1 across the theca and granulosa compartments, and by demonstrating that growth factors such as TGFB1 and EGF/FGFs affect FBN1 expression in theca cells [18]. Maylem et al. provided direct functional evidence that asprosin may enhance LH-induced androstenedione synthesis in theca cells and inhibit IGF1-mediated proliferation, positioning asprosin at the intersection of gonadotropin steroidogenesis and growth factor control of follicular expansion [18]. Batalha et al. extended their investigation into granulosa steroidogenesis, revealing dose-dependent and context-specific effects [19]. Asprosin was reported to augment oestradiol synthesis and upregulate FSHR and CYP19A1 in the presence of FSH, but under IGF1 circumstances, it resulted in reduced estradiol, elevated progesterone, and alterations in PKA and ERK signalling pathways. Maylem et al. agree with Batalha et al. in defining asprosin as a context-dependent modulator that may either augment or divert steroidogenic output, depending on whether the primary stimulus is LH, FSH, or IGF1 signalling [18,19]. Maylem et al. moreover include a stress-compatibility layer via p53 activation, as documented by Batalha et al., which may be construed as a connection between metabolic signals and cell-cycle or DNA-damage monitoring mechanisms that influence granulosa function and follicle destiny [18,19]. Maylem et al. further synthesised these patterns by comparing asprosin to other adipokines and emphasising that adipokines often display antagonistic actions in the theca and granulosa, suggesting that net follicular steroid output is an emergent property resulting from multiple signals rather than a singular ligand effect [18]. Shokrollahi et al. advocated for the integration of irisin into the “novel adipokine/myokine” paradigm, highlighting that significant mechanistic insights are obtained from non-human models, thus underscoring the need for careful translational context in discussions about IVF implications [157].

Li et al. shifted the focus to patient stratification by demonstrating that ovarian reserve status correlates with distinct intrafollicular inflammatory and adipokine profiles and by identifying IL-6 as a negative correlate of embryo quality, suggesting that cytokine-adipokine interactions may provide a vital explanatory framework for the inconsistent results observed among cohorts [65]. Barbe et al. provide molecular evidence for this connection, since adiponectin signalling is regularly linked to anti-inflammatory and antioxidant benefits [69]. This indicates that decreased adiponectin levels may promote heightened NF-κB activity, mitochondrial ROS buildup, and steroidogenic inefficacy. Estienne et al. establish a direct connection between competence, as adipokine-induced alterations in cumulus granulosa metabolism can affect pyruvate availability, redox balance, and meiotic spindle stability, consequently influencing fertilisation success without modifying traditional embryo morphological parameters [156]. Zhang et al. emphasise that single-marker methodologies may be insufficient until combined with metabolomic or multi-analyte panels that include glycolysis, lipid oxidation, amino acid turnover, and inflammatory indicators [28]. Qin et al. advocate for the multi-layer approach by illustrating that gradients between serum and follicular compartments can reveal biological significance that absolute concentrations fail to capture, particularly regarding adiponectin, and potentially applicable to irisin as proposed by Acet et al. [17]. Acet et al. and Bousmpoula et al. collectively underscore that PCOS and responder phenotype influence serum–follicular relationships for irisin, suggesting that amalgamating across aetiologies may obscure authentic biological insights [17,60]. Chang et al. also contend that BMI categories affect the interactions between adipokines and fertilisation, indicating that unique interpretative models are required for obese, non-obese, PCOS, DOR, and poor responder subgroups [13].

Takikawa et al. and Dafopoulos et al. jointly illustrate that the endocrine staging of sampling is crucial, since luteal changes and insulin-related follicular thresholds may markedly affect the signal-to-noise ratio for adiponectin and leptin [57,58]. Poretsky et al. and Stojchevski et al. assert that mechanistic studies in human granulosa cells are both viable and informative, delineating a clear translational pathway for future IVF-related research that explores dosage, timing of exposure, receptor expression, and pathway activation, rather than relying exclusively on cross-sectional correlations [88,89]. Daudon et al. and Maylem et al. demonstrate that route coupling may differ among species and cellular environments [18,90]. Therefore, future human IVF investigations should prioritise primary granulosa cell assays and, where feasible, simultaneous theca cell assays, integrating co-treatments that replicate FSH, LH, and IGF1 conditions. Batalha et al. propose that the endocrine environment can alter asprosin’s steroidogenic trajectory, suggesting that clinical investigations should evaluate asprosin alongside gonadotropins, insulin/HOMA-IR, and follicular steroid profiles to clarify associations mechanistically rather than descriptively [19]. Li et al. suggest that the incorporation of cytokines, such as IL-6, may be essential for discerning whether adipokine or myokine connections indicate metabolic support or inflammatory stress [65]. Athar et al. contend that metabolic hormones represent actionable biology rather than just correlations, therefore validating intervention studies that modify metabolic states prior to IVF and later assess follicular biomarkers as mediators rather than merely predictions [155].

Chang et al. and Qin et al. finally illustrate that adiponectin functions as a marker for fertilisation ability in certain settings. Nevertheless, its prediction accuracy declines when compartment gradients, endocrine phases, and systemic metabolic stress are not well controlled [13,56]. Acet et al. and Bousmpoula et al. establish that irisin is consistently measurable in IVF settings and correlates with insulin resistance and ovarian phenotype [17,60]. However, its predictive ability for pregnancy is inconsistent, indicating its role as a metabolic context marker with potential mechanistic significance that requires pathway-specific validation. Poretsky et al., Stojchevski et al., and Daudon et al. clarify the molecular basis for seeing irisin as an active granulosa modulator of steroidogenesis and glucose metabolism, while also warning that its effects depend on cellular context and pathway dominance [88,89,90]. Maylem et al. and Batalha et al.collectively characterise asprosin as a context-dependent modulator of theca and granulosa steroidogenesis through LH-, FSH-, IGF1-, PKA-, and ERK-associated pathways, establishing it as a potential link between fasting-like metabolic states and follicular endocrine output [18,19]. Zhang et al. and Li et al. propose a subsequent approach that combines targeted adipokine/myokine tests with inflammatory and metabolomic profiling to create a mechanistically interpretable follicular signature for oocyte competency and IVF results [23,65].

## 8. Limitations

Upon examining the existing body of data, it is crucial to acknowledge its inherent weaknesses. The bulk of clinical studies evaluating adiponectin and irisin in IVF populations are based on very small sample sizes, often consisting of less than 100 individuals, which limits statistical power and increases the likelihood of type II error. Research examining adiponectin levels in follicular fluid across individual follicles revealed correlations with fertilisation outcomes but did not consistently establish links to embryo development or pregnancy, suggesting that adipokines may influence early follicular competence rather than later implantation processes.

Secondly, there is a significant disparity in the research design, sampling methods, and patient cohorts. Certain investigations analysed follicular fluid from individual dominating follicles, whereas others used pooled samples or just assessed circulation concentrations. This disparity complicates direct comparisons across research and hinders the establishment of clinically relevant criteria. Furthermore, variations in metabolic phenotypes, including obesity, insulin resistance, and PCOS, provide additional biological variability that may influence adipokine and myokine signalling without impacting ovarian function.

The mechanistic evidence for irisin and asprosin mostly derives from in vitro granulosa cell cultures and animal investigations. These models provide significant insights into steroidogenic regulation and metabolic signalling; yet, they may not fully illustrate the interplay between the endocrine and paracrine systems inside human ovarian follicles. The absence of comprehensive human IVF trials on asprosin and the limited clinical research on irisin highlight a significant translational gap. Ultimately, the majority of research are observational and cross-sectional, complicating the ability to infer causality. Although adiponectin, irisin, and asprosin clearly influence steroidogenesis, insulin sensitivity, and follicular metabolism, it remains ambiguous whether these molecules directly determine oocyte competence or mostly reflect underlying metabolic conditions.

## 9. Future Directions

According to the review’s findings, metabolic signalling in the follicular environment should be seen as an integrated system that influences oocyte competence rather than via discrete routes. As we progress, certain pragmatic measures emerge that might bridge the divide between our understanding of molecules and their use in medicine.

The first phase should include surpassing single-marker analysis. Most research so far has examined individual adipokines or myokines in isolation; nevertheless, the findings consistently indicate the presence of a network impact. Assessing comprehensive metabolic profiles in follicular fluid may provide a more precise depiction of the functional state of the oocyte microenvironment. Mechanistically, routes linking energy balance and cellular function warrant significant investigation. The AMPK-mTOR axis, in conjunction with redox regulation, seems to represent a critical control centre. A comprehensive knowledge of how this equilibrium shifts in various metabolic contexts might facilitate targeted therapies that enhance oocyte quality.

Establishing clinically significant thresholds is also essential. Despite the correlation of several metabolic indicators with IVF results, their practical applicability is limited by the lack of defined reference values relating to fertilisation, embryo development, or implantation. Moreover, the role of asprosin in the ovary remains mostly unexplored. The predominant portion of the available data derives from other tissues, and it is unclear if the same signalling systems function in granulosa cells. Clarifying receptor expression and downstream pathways in the context of the ovaries should be a paramount goal.

From a clinical perspective, adopting integrated biomarker panels may provide more profound insights than reliance on individual indicators. Integrating metabolic, mitochondrial, and redox markers may assist physicians in more effectively stratifying patients and predicting IVF results, particularly among those with preexisting metabolic disorders.

## 10. Conclusions

Adipokines and myokines appear to regulate the follicular microenvironment by influencing mitochondrial function, metabolic signalling, and redox balance. The available evidence suggests a correlation between these pathways and oocyte competence, although most of the data is either indirect or derived from experimental models.

In this context, the integration of metabolic, mitochondrial, and redox pathways into a cohesive framework may eventually provide more precise predictive models and enhanced clinical results in assisted reproduction.

## Figures and Tables

**Table 1:** Clinical studies investigating adipokines and myokines in follicular fluid and their association with IVF outcomes.

First Author	Year	Study Type	Sample Size	Molecule Studied	Sample Type	Model	Primary Outcome	Main Findings	Strength of Evidence	Clinical Implication
Chang et al. [13]	2014	Clinical observational	67	Adiponectin, leptin	Follicular fluid	Human	Fertilisation rate	Higher adiponectin associated with improved fertilisation rate	Moderate	Adiponectin may reflect oocyte developmental competence
Qin et al. [56]	2023	Clinical observational	30	Adiponectin	Serum, follicular fluid	Human	IVF outcome	Higher serum vs. FF adiponectin in unsuccessful IVF cycles	Low	Altered adiponectin distribution may impair IVF success
Dafopoulos et al. [57]	2016	Clinical observational	20	Adiponectin, resistin	Serum	Human	Hormonal variation during IVF	Adiponectin stable during stimulation, decreased in luteal phase	Low	Adiponectin participates in metabolic adaptation during IVF
Takikawa et al. [58]	2010	Clinical observational	46	Adiponectin, leptin, insulin	Follicular fluid	Human	Pregnancy outcome	Insulin, but not adiponectin, associated with pregnancy outcome	Moderate	Metabolic factors may override adipokine effects
Li et al. [59]	2024	Clinical observational	115	Multiple adipokines	Follicular fluid	Human	Ovarian reserve	Altered adipokine profile in diminished ovarian reserve	Moderate	Adipokines linked to ovarian reserve biology
Acet et al. [17]	2016	Clinical observational	40	Irisin	Serum, follicular fluid	Human	Ovarian response	Irisin associated with insulin resistance and ovarian response	Low	Irisin reflects follicular metabolic status
Bousmpoula et al. [60]	2019	Clinical observational	140	Irisin	Serum, follicular fluid	Human	Metabolic profile (BMI)	Irisin associated with BMI and metabolic profile	Moderate	Irisin links systemic metabolism with follicular function

The levels of adipokines and myokines in follicular fluid show that the ovarian microenvironment is active metabolically. Adiponectin is linked to fertility, whereas irisin is primarily associated with metabolic status and insulin resistance. These results suggest that metabolic signalling influences oocyte competence and outcomes in IVF.

**Table 2:** Mechanistic studies examining the effects of adipokines and myokines on granulosa and theca cell function.

First Author	Model	Molecule	Target Pathway	Main Findings	Functional Effect
Poretsky et al. [88]	Human granulosa cells	Irisin	CYP19A1 regulation	Increased aromatase expression	Enhanced estradiol synthesis
Stojchevski et al. [89]	Human granulosa cells	Irisin	Multiple fertility genes	Regulation of fertility-related genes	Direct control of granulosa cell function
Daudon et al. [90]	Bovine granulosa cells	Irisin	MAPK3/1 signalling	Reduced steroidogenesis, altered glucose metabolism	Metabolic regulation of follicular cells
Maylem et al. [18]	Theca cells	Asprosin	Steroidogenesis pathways	Increased androstenedione production	Regulation of androgen synthesis
Batalha et al. [19]	Granulosa cells	Asprosin	FSHR, CYP19A1	Increased estradiol production	Enhanced granulosa steroidogenesis
Merhi et al. [91]	Human and mouse ovary	Adiponectin	AMH, kisspeptin	Regulates ovarian reserve pathways	Regulation of folliculogenesis

Adipokines and myokines directly control the metabolism and steroidogenesis of granulosa and theca cells by changing how aromatase works, how glucose is used, and how hormones signal each other. These mechanisms affect how oocytes mature and how well they can reproduce.

**Table 3:** Molecular mechanisms linking adipokines and myokines with ovarian function and oocyte competence.

Molecule	Source	Receptor	Target Pathways	Ovarian Effect	Functional Outcome
Adiponectin	Adipose tissue	AdipoR1, AdipoR2	AMPK, insulin signalling	Improved metabolic regulation	Enhanced oocyte competence
Irisin	Skeletal muscle, adipose tissue	Unknown	MAPK, metabolic pathways	Regulation of steroidogenesis	Modulates follicular metabolism
Asprosin	Adipose tissue	OR4M1	PKA, ERK signalling	Regulation of granulosa cell function	Alters steroid hormone synthesis
Leptin	Adipose tissue	Leptin receptor	JAK-STAT	Energy balance regulation	Modulates reproductive axis

Adipokines and myokines regulate ovarian function by directly influencing the metabolism, steroidogenesis, and energy-sensing pathways of granulosa and theca cells. These signalling pathways influence oocyte quality and the efficacy of IVF.

## Data Availability

Not applicable. No new data were generated or analysed in this study. This manuscript is based exclusively on previously published literature.
